# The Role of HPV in the Development of Cutaneous Squamous Cell Carcinoma—Friend or Foe?

**DOI:** 10.3390/cancers17071195

**Published:** 2025-03-31

**Authors:** Vasileios Dervenis

**Affiliations:** Department of Dermatology, St. Josef Hospital, Ruhr University Bochum, Gudrunstrasse 56, 44791 Bochum, Germany; vasileios.dervenis@klinikum-bochum.de

**Keywords:** papillomavirus, cutaneous squamous cell carcinoma, skin cancer, oncogene, commensal, immune surveillance, tumour protection, immunosuppression

## Abstract

The incidence of cutaneous squamous cell carcinoma (cSCC) is increasing, with UV radiation being the main cause. Other risk factors are age, sex, skin type and immunosuppression. Human papillomaviruses (HPVs) are associated with benign and malignant skin tumours. In contrast to anogenital and oropharyngeal carcinomas, which are caused by alpha papillomaviruses, the HPV types associated with cSCC belong to the beta-HPV genus. These viruses infect the skin epithelium and are widespread in skin samples from healthy people. It is assumed that HPV amplifies the DNA damage caused by UV radiation and disrupts the repair mechanisms of the cells, without remaining permanently detectable in the tumour tissue, the so-called hit-and-run theory. The HPV status of tumours appears to have a positive influence on prognosis and response to therapy due to increased immune infiltration, in particular by tissue-resident memory T cells and activation of immune effector cells. This favours responses to immunotherapies such as PD-1/PD-L1 inhibitors, whereas immunosuppression may promote a pro-carcinogenic effect. In conclusion, the role of beta HPV in the development of cSCC appears to be closely associated with the immune status of the host. Depending on the immune status, beta HPV can play either a protective or a tumour-promoting role, and in view of the increasing incidence of skin cancer worldwide, enhancing the immune response against virus-infected keratinocytes, e.g., through HPV vaccination, could represent a promising approach for the prevention and therapy of squamous cell carcinomas.

## Simple Summary

The incidence of skin cancer, particularly cutaneous squamous cell carcinoma (cSCC), is rising, mainly due to UV radiation. Other risk factors include age, sex, skin type and immunosuppression. Human papillomaviruses (HPVs), especially beta HPV, are linked to cSCC. These viruses are common in healthy individuals and can increase DNA damage caused by UV radiation, potentially promoting cancer. However, in healthy people, beta HPV may also trigger a protective immune response that prevents tumour formation. The role of beta HPV in cancer development depends on the immune status of the individual, acting as either protective or tumour-promoting. Given the increased rates of skin cancer, enhancing the immune response through HPV vaccination could be a promising strategy for prevention, treatment and improving responses to immunotherapies for skin cancers.

## 1. Introduction

The incidence of skin cancer, and in particular non-melanoma skin cancer (NMSC), has been increasing steadily over the past few decades and appears to be due to changes in leisure behaviour and demographic changes in industrialised countries [1]. Cutaneous squamous cell carcinoma (cSCC) is the second most common skin tumour after basal cell carcinoma (BCC) and accounts for 20% of all non-melanocytic skin tumours [2]. In immunocompetent individuals, the risk of metastasis is estimated to be around 4% [3]. CSCC often develops from precursor lesions, early in situ carcinomas, also known as actinic keratoses (AKs). The risk of progression from AK to invasive cSCC for individual lesions ranges from 0.025% to 16% per year in various studies [3,4,5]. cSCC is more likely to develop in light-exposed areas of fair-skinned people, particularly the head and neck. It is therefore clear that cumulative and excessive sun exposure is the most important etiopathogenetic factor in the development of cSCC [6]. In addition to ultraviolet (UV) radiation, a number of risk factors contribute to its development, including chronic immunosuppression (e.g., after organ transplantation), advanced age, male sex, genetic syndromes, chemical carcinogens and chronic inflammation [7]. A large multicentre European study showed a positive association between AK and the use of photosensitising drugs such as thiazide diuretics, amiodarone and diltiazem, even after adjustment to the right age [8].

UV radiation is considered to be the major factor in the development of keratinocytic tumours, while human papillomaviruses (HPVs) are increasingly being discussed as aetiological factors due to their role as viral oncogenes. HPV are DNA viruses that specifically infect the squamous epithelium of human skin and mucosa. The incidence of alpha-HPV infection varies by sex and region, with a peak during the third decade of life. A significant increase in alpha-HPV-infection-associated diseases has been observed in recent years [9,10,11,12]. The oncogenic potential of this HPV types is undisputed, and the viruses are classified into high-risk and low-risk types. Cervical carcinoma is the most common alpha-HPV-associated carcinoma, followed by carcinomas of the vagina, vulva, anus and penis as well as certain oropharyngeal carcinomas [13].

The influence of HPV, especially beta HPV, on the pathogenesis of cSCC remains the subject of controversial debate. A detailed investigation of this influence is highly relevant for several reasons: Firstly, the lack of predictive markers for the progression from AK to cSCC currently leads to the treatment of all AKs and their surroundings, which underlines the growing burden on the healthcare system and patients [14,15,16]. On the other hand, a better understanding of the pathogenetic mechanisms of HPV infection could help to identify high-risk patients and potentially enable more effective prevention and management strategies.

## 2. The Epidemiology of cSCC

The incidence of cSCC has increased worldwide in recent decades, with particularly high rates in Australia and the United States. A study estimated the incidence in Australia to be 1035 cases in men and 472 cases in women per 100,000 persons, which is among the highest in the world [17]. However, current data suggest that the incidence of cSCC in Australia is stagnating or even declining, while it remained stable in the USA between 2013 and 2015. In contrast, there was a significant increase in Germany between 2007 and 2015 [18].

Significant increases in the incidence of cSCC have also been observed in other European countries. In Finland, for example, the incidence doubled from 19 cases per 100,000 persons in 2006 to 42 cases per 100,000 persons in 2020 [19]. Similar trends have been observed in Germany, the Netherlands and Scotland, where age-standardised incidence rates increased by 2.4% to 5.7% per year. The over-60 age group has been particularly affected [20]. In the UK, the incidence of cSCC has also increased, with Wales reporting an annual increase of up to 1.6%. Iceland reported a significant increase in both in situ and invasive cSCC cases between 1981 and 2017. These trends highlight the growing burden of cSCC in developed and high-income countries [21,22]. Although cSCC incidence rates are high, mortality rates remain comparatively low and vary between countries and populations studied. A comprehensive analysis of data from 33 countries showed that the highest age-standardised mortality rates were recorded in Australia and Latvia for men and in Romania and Croatia for women [23]. A systematic review and meta-analysis by Wehner et al. found that patients with cSCC have an increased relative risk of death compared to the general population, with a relative mortality rate of 1.25 (95% confidence interval, 1.17–1.32) [24]. In addition, Schmults et al. reported that 2.1% of 985 patients with cSCC died of the disease during a median follow-up of 50 months. This study also identified risk factors for increased mortality, including tumour diameter of at least 2 cm, poor differentiation, invasion beyond fat tissue and perineural invasion [25].

## 3. Human Papillomaviruses

The papillomavirus family includes a large number of double-stranded, non-enveloped DNA viruses that are common in humans and many animals [26]. Individual virus types differ in their biological properties, such as host specificity, tissue tropism (mucosa or keratinised epithelium) and oncogenicity. In humans, 231 HPVs have been fully genetically characterised and a number of other sequences have been described that presumably represent additional HPV types (http://pave.niaid.nih.gov, accessed on 15 October 2024). HPV is divided into five genera within the Papillomaviridae family: alpha, beta, gamma, mu and nu papillomaviruses [27]. All mucosa-associated HPVs belong to alpha papillomaviruses. Based on their relative frequency in cervical cancer and the oncogenic potential, some alpha HPVs have been characterised as having a low or high risk of malignant degeneration (low-risk and high-risk HPV) [28]. Most cutaneous HPV types that infect keratinised epithelia belong to the beta and gamma papillomavirus group. In addition, some other cutaneous HPVs are classified as mu and nu papillomaviruses (http://pave.niaid.nih.gov, accessed on 15 October 2024). These HPV types are usually associated with benign epithelial lesions. Gamma, mu and nu HPV can cause common and plantar warts, whereas beta-HPV infections are usually subclinical or manifest as flat warts [29]. There is also evidence that at least some beta HPV is involved as a co-factor with UV radiation in the development of cSCC [30,31].

## 4. Frequency and Prevalence of Beta HPV

Beta HPV has been detected by PCR in over 80% of skin swabs and epilated eyebrows, with multiple beta-HPV types present in most cases [32,33]. The hair follicle is a natural reservoir. The viruses are primarily detectable in the bulge, which contains multipotent and high proliferative potential stem cells [34]. Infection with beta HPV occurs in early childhood and can persist over time. Unlike genital alpha HPV, which is mainly transmitted through sexual contact, beta HPV is transmitted through usual everyday contact with people or objects [35]. Recent findings on the frequency and distribution of HPV have come from metagenomic analyses using whole genome sequencing (WGS). In contrast to conventional amplification-based detection using consensus primers, where the spectrum of HPV detected is limited by the homology of the primers used, metagenomic analyses can detect all available HPV sequences. In a study of skin swabs from sites with different physiological characteristics (glabella, antecubital fossa, forearm, interdigital space) of healthy volunteers, beta HPV was among the 10 most common viruses [36]. As part of the Human Microbiome Project, numerous HPV sequences were identified not only in skin swab samples from healthy volunteers, but also in vaginal, oral and rectal swabs, with more than one HPV sequence detectable in most samples [37].

It appears that HPV infection or colonisation in healthy individuals is more complex than previously thought. Due to the ubiquitous nature of the spreading of beta HPV, a causal role in the development of cSCC is questionable.

## 5. Beta HPV and Association with cSCC

Beta HPV types were originally identified in skin lesions of patients with epidermodysplasia verruciformis (EV). Patients with epidermodysplasia verruciformis (EV) are particularly susceptible to HPV infection, particularly beta-HPV genotypes, due to autosomal recessive mutations in the TMC6 (EVER1) and TMC8 (EVER2) genes. Mutations in these genes result in impaired function of the CIB1–EVER1–EVER2 complex, which normally regulates the cellular immune response of keratinocytes against these viruses. These infections manifest clinically as persistent flat warts or pityriasis versicolor-like lesions [38,39,40].

In approximately 30–60% of patients, cSCC develops by the fourth decade of life in light-exposed areas where HPV5 and HPV8 are particularly detectable [30]. These two HPV types have been classified as ‘possibly carcinogenic’ by the International Agency for Research on Cancer (IARC) [41]. Subsequently, beta-HPV types were also detected in AK and cSCC in patients without EV. In the analysed studies, HPV detection was performed using PCR-based methods. However, the diagnostic approaches varied significantly, with different primers influencing the results [42,43]. Zaravinos et al. showed that HPV was significantly more common in AK and cSCC in comparison to healthy skin samples [42]. In addition, it was shown that the prevalence of HPV is higher in sun-exposed skin areas. To explain this, it was assumed that UV light has immunosuppressive effects on the one hand and on the other hand can lead to the amplification of the viral genome, which leads to an increase in viral promoter activity and a reduction in the apoptosis of infected cells [44].

While cell transformation in cervical carcinoma requires the persistence of high-risk HPVs in tumour cells and expression of viral oncogenes, beta HPVs are only detectable in very low concentrations (<<1/tumour cell) in cSCC, and the oncogenes are very weakly expressed [45]. The decreasing viral load during the progression from AK to cSCC has also been demonstrated in other studies. This supports the assumption that beta HPV may be primarily involved in the initiation of cSCC but is no longer required for the maintenance of the malignant phenotype as the disease progresses, the so-called ‘hit-and-run’ hypothesis [46,47].

## 6. What Are the Oncogenic Functions of Beta HPV?

The oncogenic properties of high-risk alpha HPV in the development of anogenital cancers are mediated primarily by the two oncogenes E6 and E7. The most important activities are the inactivation of the cellular tumour suppressor genes p53 by E6 and pRB by E7, leading to cell cycle dysregulation and inhibition of apoptosis, resulting in uncontrolled cell proliferation [48]. In most cervical carcinomas, the HPV genome is integrated into the host cell genome. The integration process is accompanied by disruption of the E2 reading frame. This blocks viral replication, which requires intact E1 and E2 proteins, and activates E6 and E7 expression, as E2 is a negative regulator of oncogene expression [49,50,51]. In addition, high-risk alpha-HPV E6 and E7 proteins interact with numerous other cellular factors that regulate cell proliferation, differentiation, immortalisation and invasiveness, as well as apoptosis, DNA repair and the immune response, thereby promoting carcinogenesis [52]. Some of these tumour-promoting activities have also been described for beta HPV, such as inhibition of apoptosis by degradation of the pro-apoptotic factor Bak, or impairment of DNA single- and double-strand breaks by interaction with XRCC-1 and p300 [53,54]. In contrast to alpha HPV, beta-HPV E6 proteins do not induce proteolytic degradation of p53. However, p53 function can be impaired by inhibition of HIPK2, which activates p53 after UV damage, thereby interfering with p53-induced induction of apoptosis after DNA damage [55]. In addition, beta-HPV E6 proteins interact with mastermind-like transcriptional coactivator 1 (MAML1), which inhibits NOTCH signalling. NOTCH is a tumour suppressor in epithelial cells that induces epidermal differentiation and regulates cell proliferation [56,57]. NOTCH, as well as Bak and XRCC-1, are activated by p53. Although beta HPVs do not directly inactivate p53, they have several mechanisms that impair p53 activity. Blocking XRCC-1, Bak and the NOTCH pathway results in cell proliferation with impaired DNA repair and apoptosis, allowing the accumulation of genomic mutations required for progression to carcinoma [30]. Another important aspect of the oncogenic effect of β-HPV is the interaction of the E6 proteins with the histone acetyltransferase p300, which normally has a stabilising function for the genome. Experimental studies show that mutations in the p300-binding domain of HPV8 E6 or the introduction of a degradation-resistant p300 protein can reverse the changes caused by E6. This highlights the central importance of this interaction for the tumourigenic effects of β-HPV [58]. These activities play a role in initiating cell transformation but are no longer required to maintain the malignant phenotype after the accumulation of further UV-induced mutations that affect the expression of tumour suppressor genes (e.g., p53, pRB)—consistent with the hit-and-run mechanism of beta HPV and UV radiation in cSCC development [30]. Co-infection with human papillomavirus (HPV) and Epstein–Barr virus (EBV) has also been shown to significantly increase the risk of developing cancer. A study by Feng et al. found a strong association between HPV and EBV co-infection and the occurrence of high-grade cervical intraepithelial neoplasia (CIN2+) in HIV-positive women. HPV and EBV co-infection has also been reported in melanoma. In addition, HPV has been observed to promote lytic reactivation of EBV in co-infected cells, which could potentially enhance EBV pathogenesis and promote oncogenic transformation and progression [59,60,61].

## 7. Protection and Benefits of Beta HPV

Recently published studies suggest that beta HPV, as part of the human skin virome, may have beneficial effects on the host. For example, beta HPV appears to induce antiviral T-cell immunity that is also directed against virus-infected dysplastic keratinocytes and may therefore prevent the development of cSCC [62]. This hypothesis is supported by animal studies. In the MmuPV1 mouse papillomavirus back skin model, virus-infected animals developed fewer skin tumours than uninfected animals after treatment with chemical carcinogens or UV exposure. The protective effect is based on the presence of certain T cells in the skin, called CD8+ tissue-resident memory T cells (TRM). Beta-HPV-induced TRM are also detectable in normal human skin and are indicative of adaptive immunity against commensal HPV infection. These epithelial TRM not only prevent the development of wart-like lesions caused by beta HPV but also suppress cSCC development by removing beta-HPV-infected cells with UV-induced cell damage [62]. The beta-HPV TRM cells appear to play an important role in tumour protection, control and prognosis, as well as response to systemic therapies in HPV-induced tumours.

TRM are a specialised subset of memory T cells that are permanently located in peripheral tissues and do not circulate in the blood. They play a critical role in local immune surveillance and enable rapid immune responses through the production of immune mediators such as IFN-γ, TNF-α and IL-2 [63]. Their ability to control viral infections is also clearly demonstrated in studies of HSV-1-infected mice. CD8+ T cells were detected in the trigeminal ganglia, where they successfully suppressed reactivation of latent virus in infected sensory neurons. This finding refutes the assumption that latent HSV-1 infections go unnoticed by the immune system [64]. CD8+ TRM cells have been identified in various tumour tissues, including melanoma and non-small cell lung cancer, and the density of the cells is associated with improved prognosis and enhanced efficacy of immunotherapies such as checkpoint inhibitors and cancer vaccines [65,66,67,68,69,70]. In mice studies, CD8+ CD69+ CD103+ TRM cells exhibited protective effects against melanoma development, while their absence increased tumour susceptibility [71].

The concept that commensal papillomavirus immunity maintains the homeostasis of highly mutated normal skin is supported by recent research. In particular, T-cell immunity against cutaneotropic papillomaviruses, such as mouse papillomavirus (MmuPV1), has been shown to play a critical role in maintaining the homeostasis of UV-damaged skin. This immune response is mediated by the infiltration of CD8+ T cells, which block the expansion of UV-induced mutant p53 clones by targeting MmuPV1 antigens expressed in keratinocytes. Mouse polyomavirus (MPyV) is often used to study polyomavirus infections in mice and can colonise the skin in a similar way to human polyomaviruses. In human studies, sun-exposed skin containing mutant p53 clones showed increased beta-HPV activity and CD8+ T-cell infiltrates compared to sun-protected skin. In addition, reduced beta-HPV RNA expression and reduced CD8+ and TRM cell infiltration were observed in AK developing from mutant p53 clones. These results suggest that the loss of HPV antigens represents an immune escape strategy that favours the expansion of mutant clones and the development of precancerous skin lesions. Beta-HPV RNA levels were significantly higher in AKs from immunocompromised patients than in immunocompetent patients. In addition, there were significantly fewer epidermal CD8+ T cells in AKs from immunocompromised patients compared to immunocompetent patients. These data suggest that the loss of HPV antigens may serve as a critical immune avoidance mechanism, facilitating the expansion of mutant clones and the development of AK. Thus, it appears that the loss of this immunity, rather than the oncogenic effect of HPV, is responsible for the increased risk of skin cancer in immunocompromised patients [72].

A meta-analysis of 814 patients with head and neck squamous cell carcinoma (HNSCC) showed a better objective response rate (ORR) and survival when treated with PD-1/PD-L1 inhibitors in HPV-positive tumours compared to HPV-negative tumours. Both Patel et al. and Wang et al. observed a trend towards significantly higher response rates in HPV-positive compared to HPV-negative tumours in patients treated with anti-PD-1/PD-L1 therapy. Patel et al. explicitly reported the tumours as alpha-HPV-associated, whereas Wang et al. reported only HPV positivity without specifying the viral genus. However, given the anatomical location, it is reasonable to assume that alpha HPV was predominantly involved [73,74]. The better response in HPV-positive tumours correlates with an increase in T-cell infiltration, activation of immune effector cells and diversity of T-cell receptors [75]. Although specific data on HPV-induced cSCC are limited, existing evidence suggests that HPV positivity may be a predictor of improved response to immunotherapy.

Studies have shown that systemic immunosuppression has an impact on the tumour microenvironment (TME) and therefore on tumour control [76]. Reduced T-cell density and impaired TRM-mediated immunity can lead to an increase in beta-HPV activity, which in combination with UV radiation has a pro-carcinogenic effect. Ineffective immunological control allows the proliferation of dysplastic or transformed keratinocytes, especially non-HPV infected cells. The selection of these cells would explain the low detection rate of HPV DNA in cSCC tumour cells [77] but does not rule out a role for HPV in the early phase of tumour development.

## 8. Beta HPV: The Oncogenic Co-Factor in Skin Tumourigenesis

The most important risk factor for the development of skin carcinomas is chronic UV exposure. In particular, cumulative sun exposure and the frequency of sunburns during childhood and adolescence are associated with an increased risk of cSCC [78]. UV radiation can be divided into two main categories: UVA (320–400 nm) and UVB (280–320 nm). The carcinogenic potential of UV radiation is primarily based on direct DNA damage caused by the formation of cyclobutane pyrimidine dimers (CPDs) and 6-4-pyrimidone photoproducts (6-4PPs). These CPDs primarily lead to C→T and CC→TT mutations, known as ‘UV-specific mutations’ [72,79]. Such mutations have also been detected in the p53 gene, leading to loss of p53-dependent cell cycle checkpoint function and uncontrolled cell proliferation [79]. UVA radiation penetrates deeper into the dermis and primarily causes indirect DNA damage. This occurs mainly through the formation of reactive oxygen species (ROS), which lead to oxidative DNA damage and promote carcinogenesis [80]. In addition, UV radiation also leads to immunosuppression, which weakens the skin’s defences. This immunomodulation is mediated in part by the upregulation of immunosuppressive cytokines such as IL-10 and the induction of regulatory T cells [79]. Individual characteristics (age, skin type and genetic predisposition), immune status and HPV infection have also been described as co-factors [78]. Evidence for an association between beta HPV and skin tumours comes from epidemiological studies, natural and transgenic mouse models and studies characterising the oncogenic mechanisms of beta HPV. Epidemiological evidence for the role of beta HPV has been described in several case-control studies showing a weak association of beta-HPV-type-specific seropositivity, DNA prevalence and viral load with cSCC [81,82,83,84,85].

Experimental animal studies support the hypothesis that beta HPV is involved in skin tumourigenesis. A particularly insightful model for this is provided by studies on *Mastomys coucha* mice infected with *Mastomys natalensis* Papillomavirus (MnPV). This virus is known to induce both benign and malignant epithelial proliferations in its natural host. Mastomys coucha mice infected with MnPV develop mostly cSCC after UV irradiation. In uninfected animals exposed to the same dose of UV, and in animals infected with MnPV but not exposed to UV, cSCC occurred only sporadically. Interestingly, MnPV was shown to affect host genome stability even in the absence of UVB exposure. In addition, expression of the viral E6/E7 proteins inhibits the repair of UVB-induced DNA damage, which further promotes tumour development. Although no Ras mutations were detected in the tumours, mutations in the Trp53 gene were common, leading to loss of function and promoting dedifferentiation of the tumours. These changes eliminate the need for continuous viral presence later in the course of the disease, as is often observed in human NMSC [86].

Transgenic mice expressing the HPV8 early region under the control of the keratin 14 promoter (to restrict expression to the stratum basale) developed cSCC and other skin lesions in the absence of physical or chemical carcinogens. This result demonstrates that HPV8 alone is sufficient to induce skin cancer in mice [87]. Silencing HPV8 E6 expression with specific siRNA delayed or eliminated papilloma development, suggesting that HPV8 gene expression is required for tumour development [88]. Another study using a transgenic mouse model investigated the influence of HPV8 on the proliferation and expansion of keratinocyte stem cells (KSCs). The authors showed that the early HPV8 gene E6 specifically stimulates Lrig1+ KSCs in the hair follicle junctional zone to proliferate. This occurs via binding of E6 to intracellular p300, which leads to activation of the STAT3 pathway and increased expression of ΔNp63. This process causes KSCs to migrate from their stem cell niche in the interfollicular epidermis to the overlying epidermis, making them more susceptible to UV-induced mutations. In AK, the immune response is thought to counteract the native infection, which may explain the increased risk of AK in immunocompromised individuals. These findings highlight the important role of the immune response in controlling HPV8-induced cellular changes. Taken together, these results and the lack of evidence for β-HPV DNA integration define the hit-and-run mechanism consistent with the role of β-HPV in the early phase of skin carcinogenesis [89].

## 9. Conclusions

**Figure 1:**
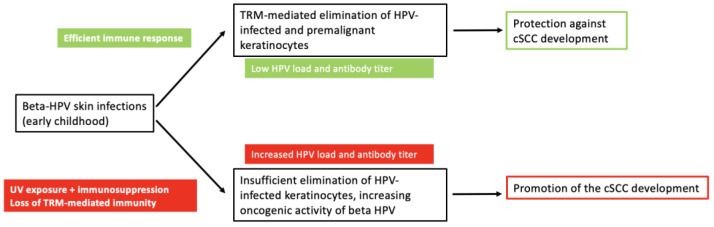
The dual role of HPV in cutaneous squamous cell carcinoma. Beta HPVs acquired in early childhood are part of the skin microbiome and persist in low concentrations. They induce cellular immunity mediated by tissue-resident memory T cells (TRM) directed against the viruses or virus-infected cells, including HPV-positive premalignant keratinocytes, thereby protecting against cSCC. UV exposure and immunological impairment increase beta-HPV load (viral concentration, number of different beta-HPV types and beta-HPV gene expression) and reduce immunological control by TRM. The increased expression of beta-HPV oncogenes, in co-operation with UV radiation, favours the development of cSCC.

Several factors are involved in the development of epithelial carcinomas. The most important risk factor for cSCC is UV radiation, which acts as a primary trigger. Infection with beta HPV and immunological factors are other co-factors in tumourigenesis, which together promote the establishment of UV-induced mutations. The role of beta-HPV infection may therefore change from tumour protection in immunocompetent individuals to tumour promotion in immunocompromised individuals (Figure 1). Regardless of the role of beta HPV as a component of the human microbiome in cSCC genesis, as a factor promoting the development of local immune functions or as an oncogenic factor for epithelial carcinomas, or both, the enhancement of immune responses against virus-infected keratinocytes, e.g., by HPV vaccination, represents a promising approach for the prevention, therapy and improvement of response to immunotherapies of epithelial carcinomas.
